# Implementing Food Traceability: Insights from Australian Red Meat and Honey Sectors

**DOI:** 10.3390/foods15091577

**Published:** 2026-05-03

**Authors:** Francesco Tacconi, Airong Zhang, Christina Maxwell, Arnold Jorge

**Affiliations:** 1 Agriculture and Food, Commonwealth Scientific and Industrial Research Organisation (CSIRO), St Lucia, Brisbane, QLD 4067, Australia; 2 Health and Biosecurity, Commonwealth Scientific and Industrial Research Organisation (CSIRO), Dutton Park, Brisbane, QLD 4102, Australia; airong.zhang@csiro.au (A.Z.); christina.maxwell@univ-poitiers.fr (C.M.); 3 Export Council of Australia, Castlereagh St, Sydney, NSW 2000, Australia; arnoldjorge@export.org.au

**Keywords:** beekeepers, biosecurity, digital maturity, digital technologies, focus groups, livestock

## Abstract

Traceability systems are increasingly central to ensure food safety, quality, biosecurity, and sustainability in agrifood supply chains. Despite advances in digital technologies, adoption and effective implementation remain uneven, with many producers still relying on paper-based systems. This study examines the motivations and conditions that enable or constrain the participation in traceability systems by Australian red meat and honey producers using the Digital Maturity Framework (DMF) as a diagnostic lens. Drawing on seven focus groups and five individual interviews involving a total of 73 producers and supply chain stakeholders from both sectors, the study investigates how value perceptions, technology and infrastructure, data analytics and management, capability, and data governance, influence producers’ engagement with traceability systems. Our findings indicate that while regulatory pressure and market opportunities incentivise adoption, several challenges persist, including high costs, limited digital skills, data sharing concerns, and sector-specific constraints. The red meat sector demonstrates higher digital maturity, driven largely by compliance mandates and an established regulatory system. In contrast, the honey bee sector exhibits more fragmented traceability adoption, challenged by the predominance of small-scale producers and limited trust in data sharing mechanisms. The comparison between two sectors reveals the influence of sectoral context. In particular, the regulatory frameworks and supply chain coordination play a relevant role in the adoption of traceability technologies. Overall, this research reveals the need for tailored policy and industry support, including regulatory harmonisation, improved data interoperability, digital infrastructure, and capacity-building initiatives to enable more consistent and broader traceability implementation across agrifood systems.

## 1. Introduction

Growing concerns about food safety and quality, and the broader impact of agricultural systems on human and environmental health have intensified demand for transparency and detailed information on food production and distribution processes [1,2]. This demand is further amplified by the increasing interdependence of global trade and markets, which generates interest not only in the food products themselves but also in the data associated with their production, movement and transformation [3,4]. In this context, traceability has become a central component of both domestic and international food supply chains.

Definitions of food traceability have evolved over time, shaped by market and regulatory requirements, and technological progress [5]. Common definitions refer to traceability as the ability to track and access information on food products’ movements and transformation throughout all stages of the supply chain [1,6]. Regulatory frameworks, such as the European Union’s General Food Law [7], further define traceability as *“The ability to trace and follow a food, feed, food-producing animal or substance intended to be, or expected to be incorporated into a food or feed, through all stages of production, processing and distribution”.* In this study, we adopt a broad definition of food traceability that also includes its use in communicating sustainability practices across the agrifood value chain.

Traceability systems serve multiple functions: ensuring compliance with food safety, biosecurity, and quality standards; guaranteeing authenticity and origin; meeting regulations and mandatory requirements for domestic and export markets; supporting producers’ efficiency in managing their operations; enabling producers to demonstrate sustainability performance and building reputation [6,8,9]. Alongside the European Union, several countries have been enacting national legislation mandating traceability in the food supply chain in recent years [9,10]. Some examples are the United States with the US Food Safety Modernization Act in 2011, Canada with the Safe Food for Canadians Regulations in 2018, China’s Food Safety law in 2015, and India with the Food Safety and Standards (Food Recall Procedure) Regulations in 2017 [9,11,12]. As a result, traceability is increasingly positioned as a core regulatory and governance mechanism within agri-food systems.

Rapid advances in digital technologies, including barcodes, QR codes, radio frequency identification (RFID), Internet of Things (IoT), blockchain and cloud-based platforms, have enhanced the capacity for real-time data collection, monitoring, storage, and sharing [5,13,14]. However, regulatory frameworks and technological availability alone do not guarantee effective or widespread adoption and implementation. Even in advanced economies, many producers continue to rely on paper-based systems due to their low cost, ease of use, and the persistence of established practices that stakeholders are reluctant to change [10,15], often at the expense of efficiency, transparency, and effective data integration [16]. These patterns suggest that besides regulation or technology availability, traceability adoption strongly relies on how systems are implemented, supported, and integrated into producers’ operational contexts.

From an implementation perspective, understanding the drivers and barriers of traceability adoption at the producer level is therefore critical. Prior research has identified a range of factors influencing traceability uptake, including implementation costs and perceived returns on investments [17,18], digital skills and capability gaps [19,20], inadequate or unreliable digital and communication infrastructure [21,22], and concerns around data governance ownership, privacy and data sharing agreements [10,14,19,23,24,25,26,27]. Across agricultural sectors, studies suggest that producers are more likely to engage with traceability systems when they perceive a clear value in terms of regulatory compliance, market access, or price premiums [28,29] and less likely to participate when traceability is perceived as costly, complex, poorly supported, or misaligned with existing farm practices [30,31]. Taken together, synthesis of this growing body of work highlights the recurring importance of value perception, capability, technology, data practices, and governance in shaping producer participation [32]. However, existing studies often focus on specific aspects of traceability adoption, such as technology acceptance, capability constraints, or data governance, while examining how these factors interact as part of a broader implementation ecosystem may provide more comprehensive insights.

The present study addresses this gap by analysing how these interactions influence traceability implementation in practice, even in the context of increasing policy and regulatory attention. To examine these factors with a holistic approach, highlighting strengths and weaknesses, this study adopts the Digital Maturity Framework (DMF) developed by Zhang and Hobman [33]. The DMF provides a diagnostic framework to assess the ecosystem supporting producers’ implementation of digital technologies, based on five interrelated key pillars: (1) Value & Culture, (2) Technology & Infrastructure, (3) Data & Analytics, (4) Capability, and (5) Data Governance [33]. In this study, we apply the DMF as an analytical tool to investigate how these pillars shape producers’ engagement in food traceability systems, and to identify key enablers and constraints affecting participation.

Australia offers an informative context for examining traceability implementation challenges, given its prioritisation on biosecurity and quality assurance systems and sustained policy attention to traceability. Despite increasing emphasis on digital transformation in Australian agriculture, technology adoption remains uneven within and across sectors [25,34]. For instance, a survey conducted by Zhang et al. [34] revealed low data collection and integration among producers, and widespread reliance on paper-based record-keeping. More recently, the Australian government launched the National Agricultural Traceability Strategy 2023–2033, investing AU$100 million to strengthen Australia’s biosecurity systems and support agricultural products by improving food traceability across the agricultural supply chain [35]. This combination of regulatory frameworks, policy investment, and uneven adoption underscores the timeliness of this research.

The objective of this study is to examine how food traceability systems are implemented in practice at the producer level, and to identify the key implementation challenges that shape their adoption and use. Using the Australian red meat and honey sectors as contrasting case studies, our research analyses how perception of value, capabilities, data governance arrangements, and supporting infrastructure interact to enable or constrain effective implementation. By comparing two sectors with different regulatory settings, practical requirements, technology, supply chain and market structures, the study identifies both shared and sector-specific challenges. Drawing on focus group discussions with value chain stakeholders and applying the DMF as an analytical lens, our findings provide practical insights for policymakers and industry bodies seeking to strengthen the implementation and effectiveness of traceability in agrifood systems.

## 2. Background

### 2.1. Australian Red Meat and Honey Bee Sectors: Context and Overview

Red meat production plays a crucial role in the Australian agrifood sector, accounting for approximately 21% of the total value of agricultural production [36]. In 2022–2023, the red meat and livestock industry generated a total turnover of approximately A$81.7 billion, with 78% of beef, veal, lamb and mutton production exported [36,37]. As a highly perishable product, it requires strict controls throughout the supply chain to ensure food safety, quality, and shelf life [38]. Inadequate handling, especially during transportation, can compromise both product safety and quality [39]. Moreover, improper production practices can raise concerns related to biosecurity, animal welfare, greenhouse gas emissions, and broader environmental degradation [40,41].

Given these risks, traceability is essential not only for ensuring product quality, but also for responding to and containing safety and biosecurity incidents, as well as for maintaining access to international markets. Hence, traceability constitutes a key component of the Australian red meat industry. A national traceability system was introduced for cattle in 1999 and later extended to sheep, goats, and pigs through the National Livestock Identification System (NLIS). Established to ensure biosecurity, food safety, and market access, the NLIS provides a framework to track the lifetime movements of individual animals across the supply chain [42]. Over twenty years later, the NLIS has consolidated a system that still allows the use of paper documents but has also driven the development and implementation of traceability technologies [43].

The Australian honey bee sector, while smaller than the red meat sector, is worth over $14 billion per year, with around 70% of honey produced from Australian native flora and approximately 10% of production exported overseas [44]. In contrast to red meat, honey is non-perishable and is widely associated with safety and health-promoting properties [45,46]. The nature of the product, combined with the predominance of small-scale operations, has resulted in fewer regulatory requirements and the absence of a mandatory national traceability framework.

The largest initiative supporting quality assurance and traceability in the sector is B-Qual and B-Trace, voluntary certification programs managed by the Australian Honey Bee Industry Council. B-Qual certifies honey producers against quality and food safety standards, offering traceability from hive to retail and aiming to uphold the integrity and marketability of Australian honey.

However, recent events have underscored the importance of strengthening traceability within the honey bee sector. For instance, the 2018 “fake honey” scandal which emerged from a study by Macquarie University that identified potential adulteration in 27% of 95 honey products sampled from Australian markets, raised major concerns about industry reputation and consumer trust [47,48]. Moreover, the recent detection of varroa mite in Australia, a destructive parasite of European honey bees (*Apis mellifera*), has highlighted significant biosecurity vulnerabilities and the need for improved traceability systems to support early detection, coordinated response, and mitigation efforts [49].

### 2.2. Analytical Framework for Traceability Adoption: The Digital Maturity Framework (DMF)

The digital maturity framework (DMF) was developed to support digital transformation in the agribusiness sector by providing a structured diagnostic, monitoring, and evaluation tool [32,50]. The framework is designed to assess producers’ “readiness” to adopt digital technologies and emphasises that technology adoption does not occur in isolation, but rather requires a broad, supportive ecosystem. This ecosystem comprises five interrelated pillars that represent the core organisational, technological, data related, capability, and governance building blocks necessary for digital transformation within agribusinesses: (1) Value and Culture, (2) Technology and Infrastructure, (3) Data and Analytics, (4) Capability, and (5) Data Governance [50]. Together, these pillars provide a holistic perspective on the conditions required to enable and sustain digital adoption across the agribusiness sector.

Given its explicit emphasis on the interplay between organisational readiness, technological capacity, and broader ecosystem enablers, the DMF offers a robust conceptual foundation for examining producers’ adoption and integration of complex digital tools. This is particularly important for traceability system adoption, which depends on the alignment of technological infrastructure [22,51], data management practices [52,53], organisational values [28,54], skills [55,56], and governance arrangements [19,57], rather than on technology alone. The DMF is therefore well suited to unpack the multifaceted factors shaping producers’ engagement with traceability technologies. Accordingly, we applied the DMF as an analytical lens to systematically examine the key factors influencing the implementation of traceability systems and technologies among Australian red meat and honey producers.

**Figure 1:**
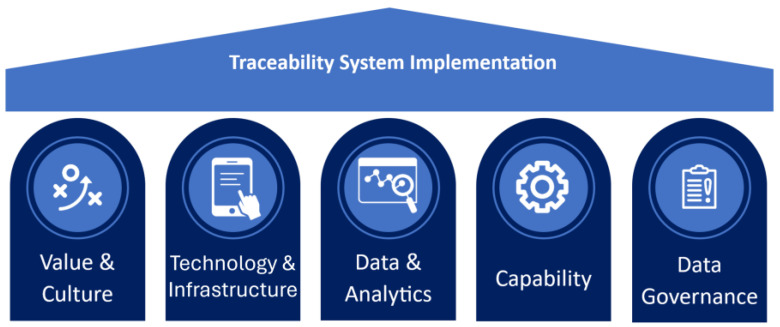
Digital Maturity Framework (DMF) application to traceability systems implementation.

In this study, the DMF was adapted to explicitly address traceability systems and technologies, with each pillar operationalised to align closely with the functional and governance requirements of traceability systems (Figure 1). Consistent with this adaptation, the five DMF pillars were translated into traceability-specific discussion themes that guided data collection, with full details provided in Section 3.1.2 and Appendix A.1. The first pillar, *Value and Culture*, captures producers’ valuation and prioritisation of the traceability systems and technologies, the perception of regulatory pressure, market incentives, benefits, as well as the extent to which the business has a clear and forward-looking traceability strategy. The second pillar, *Technology and Infrastructure*, evaluates the internal use of traceability technologies to support data collection and decision-making, along with the communication infrastructure required to collect, store, and transfer data along the supply chain. This pillar also includes the external infrastructure ecosystem and the availability of new digital traceability technologies in the marketplace. The third pillar, *Data and Analytics*, examines data quality, its use within the business, and the degree of data interoperability or the ability to seamlessly exchange and use traceability-related data across the supply chain. *Capability*, the fourth pillar, assesses producers’ knowledge, skills, and competencies to implement, manage, and effectively use a traceability system over time. Finally, the fifth pillar, *Data Governance,* includes both internal data management and data sharing protocols to maintain and store data, trust in exchanging traceability data with other actors, and the existence of appropriate formal agreements governing data ownership, privacy and use.

Together, these pillars capture both the technological and ecosystem dimensions that shape producers’ readiness and ability to adopt traceability systems and technologies and provide a structured lens for analysing traceability implementation as an integrated socio-technical system.

## 3. Methods

### 3.1. Data Collection and Analysis

This study examines the key factors influencing the adoption of traceability systems from the perspective of primary producers in the Australian red meat and honey bee sectors, using the DMF as an analytical lens. It also investigates producers’ capacity to implement traceability systems within their businesses, including their ability to collect, analyse, and share data. Primary data were collected through seven focus group discussions and five individual online interviews conducted between October and December 2023, involving a total of 73 participants. The focus group discussions were held in four Australian cities: Brisbane (Queensland), Sydney (New South Wales), Melbourne (Victoria), and Perth (Western Australia).

The research methods were approved by the CSIRO Human Research Ethics Committee (ID: 144/23).

#### 3.1.1. Participant Selection and Recruitment

To ensure a comprehensive understanding of the drivers and barriers that producers face in participating in supply chain traceability, we adopted a producer-centred, multi-stakeholder approach. This approach recognises that producers’ decisions and experiences are shaped by the broader ecosystem of supply chain partners, regulatory agencies, and technology providers. Accordingly, in addition to primary producers, we invited a diverse range of stakeholders across the red meat and honey supply chains, including technology providers, abattoirs, packers, distributors, exporters, industry bodies, and government agencies. Engaging these stakeholders enabled the research team to better contextualise producers’ views and ensured that the discussions would more comprehensively capture how other actors perceive and influence producer adoption of traceability systems.

Participants were recruited using a purposive sampling approach aimed at engaging individuals and organisations with relevant operational knowledge of traceability, while ensuring diversity in enterprise size, market orientation (e.g., direct sale vs. supermarket channels, domestic vs. export), and sectoral role. CSIRO and the Export Council of Australia (ECA) contacted key players within each sector, directly reaching out to industry organisations and producer groups in the red meat and honey sectors; targeted stakeholders involved in traceability-related activities (such as abattoirs, packers, exporters, certification bodies, and government agencies); and recognised key actors, including producers, processors, and technology providers, based on their operational involvement and experience within their respective sectors.

To ensure broader geographic representation, participants were invited from across multiple Australian regions.

#### 3.1.2. Focus Group Procedure

The research team moderated the focus groups using a semi-structured approach, centring the discussions on the five key pillars identified in the DMF (Appendix A provides a guide that was used by the research team to structure the discussion). The five pillars were addressed sequentially, and, at the end of each session, participants were also invited to bring up any additional issues related to traceability outside the framework. The same setting was adopted for each focus group to ensure consistency and comparability of data across the different sessions.

Participants were provided with participant information sheets outlining the study’s purpose, scope, and discussion topics prior to the session. All sessions were audio-recorded with consent, and research team members also took notes during the discussions as an additional source of data. Separate focus group discussions were conducted for the red meat and honey participants in each city, except for the final session in Perth, which included participants from both sectors to explore shared challenges and differences.

#### 3.1.3. Data Analysis

Audio recordings were transcribed verbatim and analysed using NVivo 14 with a qualitative content-based exploratory approach [58]. The analysis was led by the first author and structured around the five pillars of the DMF for each sector. The research team’s notes and insights were integrated into the analysis, and the results were then shared with the team for feedback and validation.

### 3.2. Final Participant Sample

A total of 73 participants contributed to the study, with between four and sixteen participants per session (Table 1). Sessions typically lasted between 1.5 and 2.5 h, depending on the number of participants and their availability.

Most producers and some other stakeholders travelled from regional areas to attend in person, while a teleconferencing option was also provided. Five participants unable to attend in person or online on the scheduled focus groups day were interviewed individually online via Microsoft Teams.

Overall, participants were actively engaged and expressed satisfaction with their involvement, appreciating the opportunity to have their say and hear perspectives from other stakeholders about traceability within their sector.

The different focus group sessions revealed consistent themes within sectors, depending on the type of agribusiness (e.g., large vs. small producers) or stakeholder role (e.g., producers, packers or exporters). This consistency across sessions indicated a strong convergence of perspectives among participants.

## 4. Results

Based on the DMF, results are presented by pillar to enable direct comparison between red meat and honey producers. Each subsection explores how participants in the two sectors reflected on a given pillar. The main findings are summarised in Table 2, which provides a comparative overview of sector specific traceability implementation patterns and key differences across the five DMF pillars.

### 4.1. Value and Culture

#### 4.1.1. Red Meat Sector

Throughout all focus group sessions, producers and stakeholders expressed a clear awareness of the value of traceability, which was generally viewed as a routine embedded in the day-to-day operations.

##### Regulation Requirements

The existing legal requirements and established systems, such as the NLIS and the National Vendor Declaration (NVD), the mandatory document required for any livestock movement along the value chain, were recognised by participants as the cornerstones for the adoption of traceability in the sector. These mechanisms were seen as indispensable for participating in both domestic and export markets:

*“If you don’t provide a level of traceability, then you don’t supply product. That’s your first look.”*

(Producer)

*“Our on-farm traceability is really strong for our transfers between properties and all this type of things, because it is obviously driven by the NLIS system.”*

(Producer)

##### Biosecurity Risk

While many participants acknowledged the administrative burden of complying with legal traceability requirements, they also recognised that without an established traceability system, producers would face significant operational risks. In particular, the threat of disease outbreaks and concerns about product deterioration during transit were identified as key risks:

*“One thing that foot and mouth and lumpy skin disease have done, being so close to our shores, is to make producers suddenly care a lot more about traceability.”*

(Processor)

*“Traceability provides us the ability to respond rapidly if there’s a biosecurity or food safety issue.”*

(Industry organisation representative)

These risks were associated with direct and costly implications, such as product loss and restricted access to export or even domestic markets, due to varying biosecurity regulations across Australian states and territories.

##### Reputation

Beyond biosecurity concerns, participants acknowledged that the lack of traceability at the individual business level could lead to indirect sector-wide implications, causing reputational damage to Australian red meat products:

*“Having traceability at national level can create credibility and simplify access to markets and reputation with customers.”*

(Processor)

This also includes potential allegations of product substitution, false provenance or other disruptions along the supply chain. In this regard, some participants saw traceability as a potential tool to be used to provide evidence and document every stage of the production process and supply chain, including insurance claims:

*“If there is an incursion here and you’ve got something […] you’ll be able to go back and trace and work out what that is, and that’s going to be like our first line of defence and to be able to mount an offensive action back again. So as a producer, it’s fundamental.”*

(Producer)

##### Market Access and Premium Prices

Participants also discussed the value of traceability in creating market opportunities, mainly from two perspectives: market access and premium prices. Market access emerged as the most significant. Without the implementation of a traceability system, producers risk exclusion from both domestic outlets (e.g., Woolworths and Coles supermarkets) and exporting companies. An industry representative confirmed market access as important by noticing an increase in demand for more detailed information on red meat products but also emphasised the importance of providing premium prices as an incentive for producers:

*“Traceability was traditionally designed around food safety and biosecurity, while now a perspective that focuses on market access is becoming more relevant. However, the producer requires a premium to provide additional information.”*

(Industry organisation representative)

Premium price opportunities were discussed especially in high-value product segments, where attributes such as organic production, carbon neutrality, or animal welfare must be verified. Processors and distributors specialising in these markets emphasised the role of transparent, verifiable data in protecting premiums:

*“Individual traceability is incredibly important from a producer client perspective […]. If at the plant we can’t verify not only where the animals come from, but that the animal in particular has been raised in accordance with the program standards, […] then that animal needs to be downgraded.”*

(Processor)

However, this link between traceability and premium prices was mostly discussed in the context of cattle production, in which producers mentioned the existence of market opportunities:

*“It doesn’t matter whether you are selling a hundred or 1000 cows, if you can prove that they’re Angus verified, you’re gonna get a premium.”*

(Producer)

According to the participants, in other livestock operations, such as lamb, goat and pig, these market opportunities from traceability were less evident if not absent. Consequently, producers in these sectors reported focusing mostly on meeting the “minimum legal requirements”.

##### Efficiency Improvements and Missed Opportunities

Finally, participants also considered the potential of implementing robust traceability systems to improve efficiency, especially when technologies support monitoring of farm production and performance. Access to downstream feedback was mostly valued by producers with vertically integrated operations, who internalise activities such as abattoirs, packing or distribution, and can then use this information to improve internal processes, livestock management, and breeding practices:

*“We’ll sort of feed the traceability information back into our genetic breeding programmes.”*

(Producer)

By contrast, when these steps were not internalised, producers reported little to no structured feedback and hence a missed opportunity to use traceability for performance improvements. This suggests a competitive advantage for larger and technologically advanced cattle enterprises compared to smaller operations or other sectors such as goat and sheep production, which may lack similar capital availability and scale. This also highlights the need for greater collaboration in non-vertical supply chains to ensure feedback is provided to producers, enabling them to capture the potential efficiency gains from traceability.

#### 4.1.2. Honey Bee Sector

Honey producers generally demonstrated a more limited awareness of the potential value of traceability, which was often considered secondary to everyday production activities and market requirements. However, emerging pressures from biosecurity risks, authenticity concerns, and niche market opportunities highlighted areas where traceability is gaining relevance.

##### Perceived Need and Regulatory Context

Compared to red meat, participants described traceability requirements in honey bee sector as less stringent. In Australia, legal requirements are generally limited to identifying the location of origin through codes assigned to individual batches, with no mandatory national traceability system. Most participants attributed this lower level of regulation to honey’s intrinsic characteristics, which present fewer food safety concerns:

*“Honey, I suppose, is a product that’s not a high-risk product like meat. You know honey, we’re so lucky! You can store it for a very long time and it’s completely safe.”*

(Producer)

This limited regulatory pressure was reflected in the low priority many producers assigned to traceability technologies, especially where current practices were perceived as sufficient for market participation. As one producer stated:

*“Traceability isn’t necessary in the beekeeper’s world. It’s only because they need to sell their honey that it takes place. […]. We’ve got sensors in our hives right now and, to me, they are useless.”*

This producer reflected the sentiment that, in this context, traceability is considered more as a market-driven requirement rather than a regulatory or efficiency one.

##### Biosecurity and Authenticity Risks

Participants acknowledged ongoing changes in the honey bee industry and the potential increase in the relevance of traceability in responding to sector-specific risks. The 2018 honey adulteration scandal and the ongoing threat posed by *varroa* were described as potential turning points, reinforcing that ensuring the authenticity and quality of honey is a priority in the sector:

*“Well, biosecurity is something that like we’re gonna be concerned about. Well, currently it’s a hot topic, obviously with varroa.”*

(Producer)

Some participants also noted that traceability could be a useful tool to document provenance and quickly localise disease incursions. This can help protect unaffected producers in the same region and prevent broader harm to the sector as a whole:

*“If something goes wrong, they can identify the sort of the area”*

(Producer)

##### Cost–Benefit Considerations and Market Opportunities

Besides biosecurity and authenticity, the perception of additional value gains from increasing traceability remained limited, especially concerning export markets. While concerns over adulteration persist among domestic consumers, a common sentiment that emerged was that Australian honey is generally considered of high quality abroad, and that confirming Australian provenance was regarded as a sufficient proof of quality by most participants. In this context, some producers argued that additional data was not necessary, and that introducing more sophisticated technological systems would represent a cost without a corresponding return to justify the investment:

*I don’t think I would [adopt technologies]. Not because it’s difficult, but I just don’t think I need it. You know, what I’m doing is…everything I need to know is there.*

(Producer)

Some exporters and boutique producers noted that implementing traceability technologies could support premium pricing and brand storytelling, especially for niche products markets:

*“That that’s a real storytelling and a real marketing tool…[…] if someone is sitting at the breakfast table in Europe and scans their honey and you know sees a map of the world come up and comes down into Australia and they go, “Yeah, I’m happy with that, that looks great.”*

(Producer)

However, others expressed concern that the cost of implementing traceability systems, particularly digital ones, was not justified by current market returns:

*“Traceability doesn’t benefit me at all. I don’t get any more money from my product.”*

(Producer)

B-Qual, the primary voluntary traceability framework in the Australian honey bee sector, was frequently raised during discussions.

Retailer and export market expectations emerged as key drivers of traceability adoption. Large domestic retailers were reported to increasingly demand traceability systems, often linked to third-party certifications like B-Qual:

*“We’re getting pressure internally inside Australia to increase traceability from the producer level.”*

(Packer)

Nevertheless, other participants described it as time-consuming and poorly incentivised. Participants noted that certain packers and retailers required B-Qual, B-Trace, or similar systems, but enforcement can be inconsistent because participation is not mandatory. For instance, one beekeeper mentioned that, in the past, during periods of low supply, some packers would also accept honey from non-certified producers, potentially undermining the perceived integrity of the system:

*“Why should I pay for audits? Why should I do all this paperwork? When times get tough, they’ll just take it from anyone.”*

(Producer)

Despite these challenges, B-Qual and B-Trace remain the main reference point for producers aiming to access major packers and retailers, and some participants expressed hope that improved incentives and clearer standards could increase uptake.

##### Variation Across Producer Types

As in the case of red meat, attitudes towards traceability varied across different types of honey producers, shaped by their scale of operations, market orientation and business models.

For instance, the size and type of business, as well as the commercialisation strategy created a significant distinction between (1) conventional beekeepers, typically operating on a large- or medium-scale and selling to supermarkets and large packers; (2) emerging boutique producers, often small-scale and vertically integrated, selling directly to consumers online or in small shops; and (3) hobbyist or recreational beekeepers, managing only a few hives and selling at farm gates.

Conventional beekeepers seemed to prioritise the minimal traceability requirements necessary to comply with supermarket and packer standards. Boutique producers, by contrast, placed greater value on investing in traceability technologies (e.g., QR code) for market differentiation, recognising the opportunity to convey the narrative behind their operations and products as a means of adding value. This was particularly the case for small beekeeping operations targeting niche markets and producing premium honey varieties (e.g., Jarrah, leatherwood, manuka or organic). Hobbyist or recreational beekeepers perceived limited value in traceability, as their small-scale, farm-gate sales do not involve large-scale markets or formal distribution requirements.

### 4.2. Technology and Infrastructure

#### 4.2.1. Red Meat Sector

Participants reported that a range of new traceability technologies is emerging in the red meat sector. At the same time, frequent changes in domestic and export regulations, combined with variations across states and markets, create uncertainty for producers about which technologies are most worthwhile to invest in.

Among the various types of red meat production, cattle emerged as the most mature and advanced in traceability technologies, while other sectors such as lamb and goat, noted that digital solutions (e.g., ear tags) are often not designed for their specific needs and would require further innovation tailored to their operations.

Another distinction emerged within the red meat sector between early adopters and large commercial operations versus smaller-scale or hobby producers. The cost of implementing traceability through advanced digital solutions was raised as a key discriminant, as larger operations had more investment capacity. As a result, the use of non-digital methods, such as paper documents, to record and exchange traceability information along the supply chain is still common, confirming the findings of previous studies [34]:

*“In general, I got most of meat transfers certificates which are still a piece of paper.”*

(Exporter)

Processors and exporters also discussed this issue, noting that working with paper-based documents often leads to a higher risk of mistakes during data entry and is more time-consuming compared to digital records.

Finally, access to the internet in rural areas was identified as a major barrier to the use of traceability technology:

*“One of the biggest challenges that we have as a beef producing nation is that a lot of our producers are situated in regional, remote and rural areas that often don’t have the best connectivity.”*

(Industry organisation representative)

Satellite internet or alternative connectivity methods (e.g., additional SIMs) were mentioned as potential solutions, although some participants raised the issue of additional associated costs. In this regard, applications working offline were suggested as the most practical short-term alternative response available. These applications allow users to record data and upload it once back online. Despite this advantage, participants considered offline entry only as a second-best option, as it did not allow for real-time information traceability:

*“If there is an incident or something going wrong with the animal, if you don’t have coverage, you can go there with your tablet and go back to the main office, but it’s too late. So sometimes you really wanna have instant information.”*

(Industry organisation representative)

Poor network coverage also compromises traceability during transport, creating potential liabilities for both domestic and international trade, particularly when instant access to refrigeration or storage data is required.

#### 4.2.2. Honey Bee Sector

While some early adopters participated in the focus groups, the discussions highlighted that paper-based traceability remains widespread in the honey bee sector, with relatively low uptake of digital solutions. Technology providers confirmed that digital solutions are already available for beekeepers and under further development. However, most producers saw little incentive to invest in digital systems.

B-Qual, the main voluntary scheme available, requires structured record-keeping, but still allows either paper-based or digital records, with verification conducted through audits that accept both formats. Some packers were reported to pay premiums for the honey certified under these programs. Yet, the adoption of digital solutions remains limited, particularly among smaller producers. Some beekeepers noted that the extent and consistency of these incentives were often not sufficient to justify the additional costs and skills required for implementation:

*“It’s also about costing. To build these systems, it costs money. And if the bottom player doesn’t get anything out of it, they do not see a value. There has to be, should be, a subsidy and some description on how to use the technology.”*

(Producer)

Commonly cited barriers included the cost, complexity of implementation, and unfamiliarity with the use of digital technologies. Poor coverage was also raised, especially by beekeepers placing hives in national parks or reserves. Using software allowing for offline upload was frequently indicated as a potential solution during the discussions. Nevertheless, many continue to rely on informal methods, such as hand-written notes or even memory, later transferring the information into spreadsheets:

*“You are all sticky and wet and you’re tired and with bee stings and yeah, things all over you and all the rest of it. And you’re trying to write it in a book. You know we’ve got good memories beekeepers. And then we get home, we write it.”*

(Producer)

Most beekeepers reported preferring paper due to established habits and its simplicity. At the same time, some acknowledged that technologies could help reduce labour and improve their work–life balance but would only consider investing if the solutions were more affordable and user-friendly. An example cited was the use of sensors to measure and keep track of the load of honey in each hive, which could substantially reduce the workload of manually checking the hives over time.

### 4.3. Data and Analytics

#### 4.3.1. Red Meat Sector

In the red meat sector, producers generally did not see the data collection process as a major issue. Instead, most of the discussions focused on the complexity of meeting diverse domestic and export requirements. Participants highlighted inconsistent regulations across different jurisdictions, which complicate compliance and create inefficiencies:

*“One of the biggest challenges is that we have inconsistent legislation and regulation between the jurisdictions as to what’s required, how things are dealt with.”*

(Industry organisation representative)

This fragmentation, combined with increasing demands from supermarkets, exporters, and regulatory bodies, has underscored the need of simplification and standardisation as key priorities to support traceability adoption:

*“There’s too many pieces of information that are needed on a National Vendor Declaration that are potentially unnecessary now, and so we need to simplify that process.”*

(Processor)

Additionally, significant attention was given to the low interoperability between data platforms and different stages in the value chain. Limited integration reduces the capacity for analysis and feedback, limiting opportunities for producers to capture added value from traceability. Larger or vertically integrated operations, with both the expertise and resources to collect and process data beyond NLIS minimum requirements, were better positioned in this context. This suggests that the advantage lies not in the technology itself but in the broader business-specific characteristics, including capabilities, which links directly to the next pillar.

#### 4.3.2. Honey Bee Sector

In the honey bee sector, the traceability data required from beekeepers are generally limited to the provenance of the honey, as this information is crucial for tracing potential pest outbreaks and ensuring transparency about the product’s origin. However, unlike red meat producers, most Australian beekeepers do not own the land where their hives are placed. They either collaborate with other farmers for pollination services or locate hives in favourable locations in national parks, conservation areas, and reserves under legal permits. Hence, the actual location of the hives (e.g., coordinates) is considered and usually kept confidential, especially from competitors:

*“I think the biggest issue that we face with traceability is other beekeepers.”*

(Producer)

As a result, traceability records typically include only the postcode of the nearest council, along with batch numbers and harvest dates. During the discussions, participants expressed reluctance to provide more detailed operational data, mentioning both competitive concerns and the small-scale nature of their businesses. Some beekeepers maintain additional internal records, such as hive productivity for each location and season, but primarily for personal management rather than external reporting:

*“So, I keep very detailed records and all the honey that I sell, where if it’s my honey, it’s got a batch number on it which is the date that I extracted. […] I do it for myself. You know, I can look back at some stage in the future.”*

(Producer)

*“We can work out the productivity of each hive. So, if there’s a problem, then you can go back. We can also trace back where the sites are, so you know which are good sites in which seasons, so it’s a business tool.”*

(Producer)

### 4.4. Capability

#### 4.4.1. Red Meat Sector

Despite the availability of technologies, participants discussed considerable differences in capabilities within the red meat sector. Some “early adopters” among the participants demonstrated extensive knowledge of traceability requirements and available technologies. In contrast, processors and exporters remarked that the ability and interest to implement and manage traceability technologies can vary substantially across producers. Differences were particularly evident again between large commercial operations and smaller or hobby farms, as well as between older and younger generations of farmers:

*“It varies from farm to farm, like really drastically. And bigger businesses are much more likely to [have the necessary skills]. There’s definitely variation in age.”*

(Processor)

Business scale and age were identified as key factors influencing technological capability, but participants also mentioned challenges in accessing skilled labour to manage and analyse the data collected:

*“We’re lacking skills in terms of interpreting that data. So, we’re having to look for people to actually fill those gaps because we’re getting good quality data, but we’re not using enough of it.”*

(Producer)

These capability limitations reduce producers’ ability to efficiently use traceability information, which significantly lowers the potential benefits and, consequently, diminishes their motivation to adopt new digital solutions.

#### 4.4.2. Honey Bee Sector

In the honey bee sector, participants emphasised that traceability within the honey bee sector is mostly characterised by paper-based recording and a low average level of digital literacy:

*“The technology is out there if you want it, but it’s a matter of getting beekeepers to actually do something about it, because I’m suspecting the record keeping of a lot of beekeepers is very very woeful.”*

(Producer and technology provider)

Beyond digital skills, some participants stressed that improving beekeepers’ awareness and understanding of the opportunities linked to implementing traceability systems should be a priority for encouraging greater engagement:

*“I know there’s a lot of benefit, but beekeepers don’t know. So, beekeepers need to know the benefit of traceability and good food culture.”*

(Producer)

Additionally, the elevated average age of beekeepers in Australia was identified as amongst the major factors hindering innovation within the sector:

*“If you want to get older generation doing it too, the technology will be the problem part. Yeah, they can’t do the technology part.”*

(Producer)

Another concern raised by participants was the rapid growth of recreational beekeeping. In this context, hobbyist beekeepers were viewed as particularly unlikely to adopt traceability technology or systems, as their direct-to-consumer sales typically bypass formal supply chain intermediaries. Conversely, packers noted encouraging signals about technology uptake among younger generations of beekeepers, who appear more aware of the benefits of sharing information to promote premium honey varieties and engage customers through storytelling. This observation was supported echoed by the boutique honey producers in the focus groups, who demonstrated strong interest and active engagement in digital innovations.

### 4.5. Data Governance

#### 4.5.1. Red Meat Sector

Data governance was mostly discussed in relation to data ownership, data sharing, and access to feedback. In general, participants agreed that red meat producers were willing to share the information required under traceability regulations:

*“Sharing data at the moment is the best way to move the industry forward, so most of the Breeders in our association will happily share data to help improve and uplift the industry.”*

(Industry organisation representative)

However, participants highlighted the need for greater transparency about data ownership and the subsequent uses of the data:

*“I do think that needs to be said who actually owns that data and so on. As a farmer, I want to own that data.”*

(Producer)

Participants also stressed that data sharing should be a two-way process, enabling producers to access and make some use of the information from later stages in the supply chain. At present, participants perceive data sharing mostly as a one-way flow from producers to the next supply chain partners. Vertically integrated operations, which control multiple stages from production to commercialisation, were noted as exceptions having broader ownership and access to data.

#### 4.5.2. Honey Bee Sector

Together with “Value and culture”, data governance emerged as a prominent pillar, with participants dedicating a considerable attention to the discussion of this topic. As noted in Section 4.3.2, the exact location of hives can represent commercially sensitive information in the beekeeping sector, as it is strategically important and beekeepers are generally unwilling to disclose it. Packers and exporters observed that building trust with beekeepers is therefore essential to establish business relationships and secure data-sharing agreements:

*“Traceability has fundamental issues in the beekeeping industry because the beekeeper actually does not want anyone else to know where those bees are. That’s the fundamental problem that we face in this particular industry.”*

(Packer)

These concerns over data privacy are reinforced by the limited regulatory framework. Unlike the beef industry, where mandatory systems have established clear governance structures for traceability data, honey producers are reluctant to share additional information voluntarily:

*“In the beef industry they are seeing it as a ‘Well, we have to do this, we may as well embrace the technology’. Whereas beekeepers are saying: ‘Well, we don’t really have to do this, cause no one’s making us.”*

(Producer)

In this regard, some producers expressed hesitation about the introduction of stricter legal requirements:

*“I get very worried about regulatory capture, as the unintended consequence of putting more regulation on is actually not a better result.”*

(Producer)

Nevertheless, many participants, especially producers with a traceability system already in place, recognised that regulation could play an important role in fostering trust and encouraging wider adoption of traceability and related technologies.

## 5. Discussion

This study explored the drivers and barriers that enable or constrain the implementation of traceability systems among Australian red meat and honey producers through the lens of the DMF. By comparing two contrasting sectors, our findings highlight both sector-specific dynamics and broader patterns that can inform policy, industry strategies, and technology design. Our findings build on prior literature emphasising the importance of perceived value, technology, data management, capability, data governance and the supporting ecosystem overall in traceability adoption [29,32,55]. The DMF proved to be effective in guiding the discussions and analysis systematically, capturing comparable insights across the five pillars.

### 5.1. Value and Culture: Regulation vs. Market-Driven Motivation

Red meat producers demonstrated a stronger motivation for traceability, primarily driven by regulatory compliance, biosecurity risks, and reputational concerns. This confirms findings from Hernandez-Jover et al. [56], Huang & Fu [29], and Corallo, Latino [59] that regulation and perceived usefulness strongly influence adoption. Established systems such as NLIS and NVD provide clear legal requirements and a structured framework, making traceability a day-to-day practice. Differences were also observed within the red meat sector, with cattle producers benefiting from a longer regulatory history and greater market share, whereas goat and lamb operations tend to be less technologically advanced and mostly use traceability for minimum compliance. Participants highlighted that market access and premium pricing further incentivise adoption, particularly for high-value cattle operations, emphasising that perceived benefits can drive adoption decisions [17,60,61]. Red meat producers also stressed that receiving feedback information from other supply chain actors could enhance perceived value from traceability, suggesting that data use and sharing mechanisms are critical to encourage further engagement.

In contrast, honey producers showed lower acceptance and motivations towards traceability adoption. With limited regulatory requirements, traceability was generally not seen as a priority but was instead driven by voluntary certifications and niche market opportunities. This finding aligns with literature showing that voluntary traceability adoption is primarily influenced by perceived usefulness, market incentives, and branding opportunities rather than compliance obligations [28,29,62]. Boutique and export-oriented honey producers were more likely to adopt digital traceability for premium marketing, whereas hobbyist and conventional producers showed limited interest, revealing heterogeneous beliefs within the sector [31]. Nevertheless, the focus group discussions suggested that the honey bee sector is undergoing gradual transformation, with emerging actors demonstrating greater digital literacy and a more proactive stance towards adopting traceability technologies.

### 5.2. Technology and Infrastructure: Access and Usability

Our study indicates that paper-based traceability systems still represent a frequent scenario for both sectors, confirming previous research conducted in Australia [25,34,63].

The red meat sector benefits from more mature technological solutions, yet challenges remain related to cost, connectivity, and suitability across different types of operations. Due to disparities in investment capacity and digital literacy, larger and vertically integrated enterprises were better positioned to implement digital traceability solutions, whereas smaller operations tend to rely on paper-based records. This confirms prior findings on the existing digital divide in agriculture [10,34,56]. Poor internet connectivity in rural areas further complicates the reliance on traceability technologies, limiting the possibility to receive real-time information [52,55].

Similarly, the use of paper-based records is still predominant in the honey bee sector, with limited digital uptake, mostly driven by boutique producers or early adopters. Costs, complexity, and lack of clear incentives emerged as the main barriers. These results confirm earlier findings that ease of use, perceived effort, and, mostly, cost-effectiveness to justify the initial investments strongly influence adoption, especially in small-scale operations [14,15,22,64].

### 5.3. Data and Analytics: Interoperability and Data Quality

In the red meat sector, producers highlighted challenges related to inconsistent standards and regulations with low interoperability among platforms, creating uncertainty about requirements and further reluctance to invest in technology. These observations are consistent with prior studies emphasising that interoperability and data management are critical for realising benefits from digital traceability [19,21,53,55]. On the other hand, vertically integrated operations showing appropriate skills and infrastructure to capture value from analytics, reinforced that digital maturity heavily depends on technological and organisational capabilities [55].

Data collection for honey producers primarily focused on batch identification and provenance. Operational data such as hive location or productivity were often kept private due to competitive concerns, highlighting trust and data ownership as key determinants of voluntary adoption [23,54,57]. These findings suggest that data sharing concerns can limit adoption and broader system integration, even when potential market opportunities are available.

### 5.4. Capability: Skills and Knowledge Gaps

The discussions around capabilities highlighted differences both between and within the sectors. In line with previous studies [21,65], variability in skills was particularly pronounced between large commercial operations and small producers in the red meat sector. Smaller farms exhibited limited digital literacy, affecting the ability to leverage digital tools fully, and consequently, their motivation to adopt, a pattern also observed in previous research [17,30,56].

In the honey bee sector, capability gaps were more evident due to small-scale operations and high average age among commercial and hobbyist beekeepers. Awareness of traceability benefits was limited, suggesting targeted education and support programs could enhance adoption, especially for younger and digitally literate producers. These findings also align with recent studies [29,62]. Nevertheless, boutique and export-oriented operators demonstrated proactive adoption of technology and greater digital literacy, highlighting within-sector heterogeneity.

### 5.5. Data Governance: Trust and Ownership

Trust and data governance emerged as a critical pillar, particularly in the honey bee sector. While red meat producers generally accepted regulatory data-sharing requirements, concerns over ownership and the limited feedback information returned from supply chain partners emerged from the discussion. These points align with prior findings on the importance of clear data-sharing protocols [19,21]. Participants from vertically integrated operations reported benefiting from greater control over data, strengthening competitive advantages. Hence, enhancing governance in this area may increase producers’ perceived value of traceability systems, incentivising further engagement and technology adoption.

In the honey bee sector, producers’ reluctance to disclose hive locations and other operational data confirms the central role of trust, as suggested in previous studies [20,54,57,66]. Despite the existing voluntary certification schemes, measures and regulatory frameworks addressing data privacy and ownership concerns should be a priority to encourage traceability adoption in the honey bee sector.

### 5.6. Implications, Limitations and Future Research

Our results suggest that effective traceability adoption requires coordinated policy frameworks, targeted technological support, and tailored capability-building initiatives that reflect the distinct characteristics of each agricultural sector.

In the red meat sector, simplifying compliance and enhancing interoperability could improve efficiency, whereas for honey, incentives, increasing awareness, and trust-building may be more effective. In both cases, improved connectivity and access to user-friendly digital tools are essential to narrowing the digital divide, simplifying the implementation of traceability systems, and broadening access to the benefits they generate, thereby enhancing producer perceived value and motivation to adopt. While clear economic benefits and market advantages are key to encourage investment, the establishment of transparent protocols and trusted systems for data sharing, ownership, and use should be prioritised to mitigate perceived risks and enhance participation, particularly in sectors with sensitive operational data. Finally, targeted programs addressing digital literacy and awareness of traceability benefits can bridge capability gaps, particularly among older and smaller-scale producers.

The application of the DMF provided a robust analytical framework to systematically capture cross-sectoral contrasts and sector-specific barriers, highlighting the interplay across the five pillars. By structuring the analysis around these pillars, the framework allowed for consistent comparisons between the red meat and honey bee sectors and to develop policy and industry recommendations. Future research may extend the application of the DMF through broader surveys to quantify adoption patterns and assess the impact of new regulations, technology solutions, or incentives on uptake. Another interesting area of investigation could be comparative studies across additional sectors or countries to extend the understanding of the role of the supporting ecosystem in traceability adoption.

While the focus groups captured a diverse range of producers and other supply chain stakeholders from different Australian regions, the scope of the study is at national scale. This provides a comprehensive overview at the national level but should be considered when interpreting the findings, particularly in terms of their transferability to specific regions, producer groups, or regulatory settings. To address this limitation, future research may focus on specific regional context, production systems, or producer profiles to generate more nuanced insights and identify more context- or farm-specific issues.

## 6. Conclusions

This study used stakeholder focus group discussions guided by the DMF to examine the adoption of traceability systems in the Australian red meat and honey bee sectors.

By discussing the DMF five pillars, namely Value and Culture, Technology and Infrastructure, Data and Analytics, Capability, and Data Governance, participants addressed sector-specific drivers, barriers, and opportunities for traceability adoption. In the red meat sector, adoption is largely driven by regulatory compliance, biosecurity considerations, and market access, with larger and vertically integrated operations benefiting from greater technological capacity and data integration. In contrast, honey producers operate in a predominantly voluntary, market-driven context, where adoption is mostly influenced by niche market opportunities. Across both sectors, scale, resources availability, digital literacy, and trust emerged as key determinants of readiness and capacity to implement traceability systems.

These findings emphasise that digital traceability adoption is shaped not only by technology, but also by regulatory frameworks, market structures, organizational capabilities, and producer characteristics. Hence, tailored strategies are key to promoting and supporting wider adoption. The DMF proved to be an effective diagnostic and comparative tool, showing how the interactions between the pillars can influence producer motivation, and providing a framework that future research can apply to extend this work to additional sectors, regions, or countries, and by combining qualitative insights with larger-scale quantitative studies to examine variation in traceability adoption. Such research would further support the development of targeted policy and industry interventions tailored to specific production contexts.

## Figures and Tables

**Table 1:** Number of participants and their role in the value chain by sector.

Red Meat	n	Honey Bee	n
Producer	10	Producer	13
Technology provider	9	Producer and industry org. representative	9
Industry organisation representative	7	Industry organisation representative	2
Processor	5	Packing and distribution	5
Exporter	3	Government agencies	3
Audit and certification company	1	Audit and certification company	2
Government agencies	1	Technology provider	2
Research company	1		
**Total**	**37**	**Total**	**36**

**Table 2:** Comparative summary of traceability implementation patterns and sectoral differences across the Digital Maturity Framework pillars.

DMF Pillar	Red Meat	Honey Bee	Key Insights/Differences
Value & Culture	Highly integrated in routine activities; strong regulatory and market drivers.	Emerging awareness; minimum requirements; market-driven, in particular for premium producers.	Red meat is more established; while in the honey bee sector adoption varies by producer type.
Technology & Infrastructure	Moderate-high adoption for cattle, lower for other livestock operations; digital divide across operation size.	Mostly paper-based; low digital adoption; early adopters in boutique producers.	Infrastructure barriers (connectivity, costs) impact both sectors.
Data & Analytics	Sufficient for compliance; standardisation/interoperability issues.	Limited data collection; location sensitive; in-business management.	Red meat benefits from feedback loops; honey bee sector is limited by confidentiality and small scale.
Capability	Large operations developing digital literacy; small/hobby farms still limited.	Generally low digital literacy; younger boutique producers more engaged.	Low awareness and digital literacy affect adoption across both sectors.
Data Governance	Willing to share; want transparency and feedback.	Lower trust; sensitive information (hive locations).	Red meat considers standardisation a priority while honey bee sector considers governance and trust as critical.

## Data Availability

The data used in this article are not readily available due to confidentiality and the risk of participant re-identification. Requests to access the datasets should be directed to the corresponding author.

## Appendix A. Discussion Points Provided to Participants

This appendix includes the document shared with participants prior to the focus group sessions and interviews. It was used by moderators as a guide to structure the discussions around the five pillars of the Digital Maturity Framework (DMF).

### Appendix A.1. Roundtable Discussion

The National Agricultural Traceability Strategy 2023 to 2033 aims to strengthen Australia’s biosecurity systems and support agricultural products and value-adding benefits domestically and internationally. However, the implementation of the Strategy relies on producers’ active participation and uptake.

Funded by DAFF’s Traceability grant, this research project aims to understand the areas of strength and challenges in implementing traceability systems at agribusiness level. The workshop discussion will focus on the supporting ecosystem and include the following aspects:

1. **Value and culture**

To adopt traceability system into business operation, it relies on agribusiness places a high priority and value on traceability system. Discussion points:

- How do producers see the value of implementing traceability?
- How do their supply chain partners see the value of implementing traceability?
  ○ Are they aligned?

2. **Information systems and infrastructure**

To support the streamlined collection, extraction, and transfer of required data for traceability along the supply chain, relevant information systems, technologies and procedures are needed both within agribusiness and between supply chain partners. The agribusiness’ communication infrastructure also needs to fully support its data and digital technology needs. Discussion points:

- The current status of agribusinesses’ information systems technologies and infrastructure in fulfill the purpose of streamlined collection, extraction, and transfer of required data for traceability.
- The challenges agribusinesses face on this front.

3. **Data and analytics**

To implement traceability system, agribusiness needs to collect all relevant data that are of high quality, and the data can be easily accessed in-business and through the supply chain. Discussion points:

- The current status of agribusiness’ data collection and data use, and
- Data interoperability across the supply chain,
- The challenges agribusinesses face on this front.

4. **Capability**

To implement traceability system, agribusiness needs the knowledge, skills and abilities in working with digital technologies and data for the function of traceability system. Discussion will focus on:

- Current level of knowledge, skills, abilities and awareness of traceability technologies as well as data collection and data use.
- Challenges on this front? (e.g., access to information, lack of skilled labour, capacity to train employees, external support)

5. **Data governance**

Data Governance is about data management and data sharing to ensure the integrity and security of data.

- Agribusiness’ awareness and adoption of data management protocols/systems/allocated staff to efficiently and securely maintain and store data,
- Agribusiness’s concerns over data sharing, trust in other supply chain actors to misuse and abuse their data.
- How the data sharing between businesses are governed by agreements for appropriate use.

6. **Any other challenges**
